# Surface Dependent Inhibition of Mycobacterium abscessus by Diverse Pseudomonas aeruginosa Strains

**DOI:** 10.1128/spectrum.02471-22

**Published:** 2022-11-17

**Authors:** Ayantu W. Idosa, Daniel J. Wozniak, Luanne Hall-Stoodley

**Affiliations:** a Department of Microbial Infection and Immunity, The Ohio State University, Columbus, Ohio, USA; b Department of Microbiology, The Ohio State University, Columbus, Ohio, USA; University of Maryland School of Pharmacy

**Keywords:** *Pseudomonas aeruginosa*, *Mycobacterium abscessus*, interbacterial interaction, antagonism, biofilms, bacteria-bacteria interactions, cell-cell interaction, cystic fibrosis pathogens, nontuberculous mycobacteria

## Abstract

Both Pseudomonas aeruginosa and Mycobacterium abscessus are bacteria that cause pulmonary infection in people with inflammatory lung disease, including individuals with cystic fibrosis (CF). These bacterial species inhabit the same environmental reservoirs (soil and water) and can be coisolated in the lungs of people with CF. We investigated the interaction of these bacteria and found an antagonistic interaction favoring P. aeruginosa that was observed in biofilms but not in planktonic cultures. This antagonism extended to multiple P. aeruginosa strains and against Mycobacterium smegmatis. We tested known P. aeruginosa mutants for genes that can play roles in interbacterial contact-dependent (type III and type VI secretion systems) and contact-independent (quorum sensing, type II secretion) antagonism pathways to interrogate the mechanism of action. Our results indicate that well-known mechanisms of interbacterial competition are not responsible for the antagonism of P. aeruginosa toward M. abscessus, suggesting a novel antibacterial strategy.

**IMPORTANCE** The biofilm lifestyle is favored by many organisms, and understanding interbacterial interactions that occur between coisolated bacterial species can provide new information regarding bacterial defense mechanisms and antibacterial targets. This may also provide insights into possible interbacterial interactions impacting host immunity during coinfection. Here, we investigate an antagonistic interaction favoring P. aeruginosa over M. abscessus exclusively in dual-species biofilms and not in liquid coculture.

## INTRODUCTION

Many bacterial species are found in diverse communities and can be coisolated from various infections. These various bacterial species can be found in biofilms, which can be defined as a community of microbial cells that are enclosed in a polymeric extracellular matrix. Different types of interactions can take place in these communities that are either beneficial or detrimental to the survival of the bacterial species of interest. This study focuses on the interaction between Pseudomonas aeruginosa and Mycobacterium abscessus, which are pathogens that can be found in the same environmental reservoirs, including water sources and soil (1, 2), and are coisolated from various pulmonary infections, including in people with cystic fibrosis (pwCF).

P. aeruginosa, a Gram-negative opportunistic pathogen, is known to cause nosocomial infections and is commonly isolated in pwCF (3). M. abscessus, a rapidly growing nontuberculous *Mycobacterium* (NTM), has also shown an increased prevalence in chronic lung infections in pwCF (4, 5). M. abscessus has two morphotypes, rough and smooth, which are mainly distinguished by the presence of glycopeptidolipids (GPL) in the cell wall of the smooth and absence of GPL in the rough. Both morphotypes can survive intracellularly in macrophages, and both can form biofilms (6).

CF is an autosomal recessive genetic disease that leads to progressive lung failure and chronic disorders in other organs of the body, including the pancreas and the liver (7). The reduced mucociliary clearance in the lung due to mutation of CFTR can sustain bacterial infections, which can develop into biofilms over time making them more challenging to treat (8, 9).

Notably, P. aeruginosa and M. abscessus are both found in the same environmental reservoirs (1, 2). Previous studies have shown that M. abscessus had specific genes common to P. aeruginosa that are thought to have been transferred through horizontal gene transfer from distantly related environmental bacteria (10). Both organisms have also been coisolated from pwCF and other respiratory infections, including studies showing that NTM-positive pwCF are more likely to be infected with P. aeruginosa (11–14). Case studies of severe pulmonary infections of M. abscessus and P. aeruginosa coinfection in patients with pneumonia have also been reported (15).

There is limited understanding, however, of the interaction between these two important opportunistic pathogens. One study suggested that M. abscessus can degrade Pseudomonas quinolone signal (PQS), which is a quorum-sensing (QS) molecule (16). This finding was compelling, as P. aeruginosa is equipped with multiple virulence factors, including various secretion systems and secondary metabolites, allowing it to have a survival advantage over other microbes in cocultures, including biofilms (17, 18). Here, we examine the interaction between these two bacterial species and report that P. aeruginosa can antagonize M. abscessus in colony biofilms (19) but not in liquid coculture. Furthermore, we report that this antagonism does not appear to be mediated by known mechanisms of P. aeruginosa interbacterial antagonism, suggesting a novel antibacterial strategy.

## RESULTS

### P. aeruginosa-mediated antagonism of M. abscessus in biofilms.

**FIG 1:**
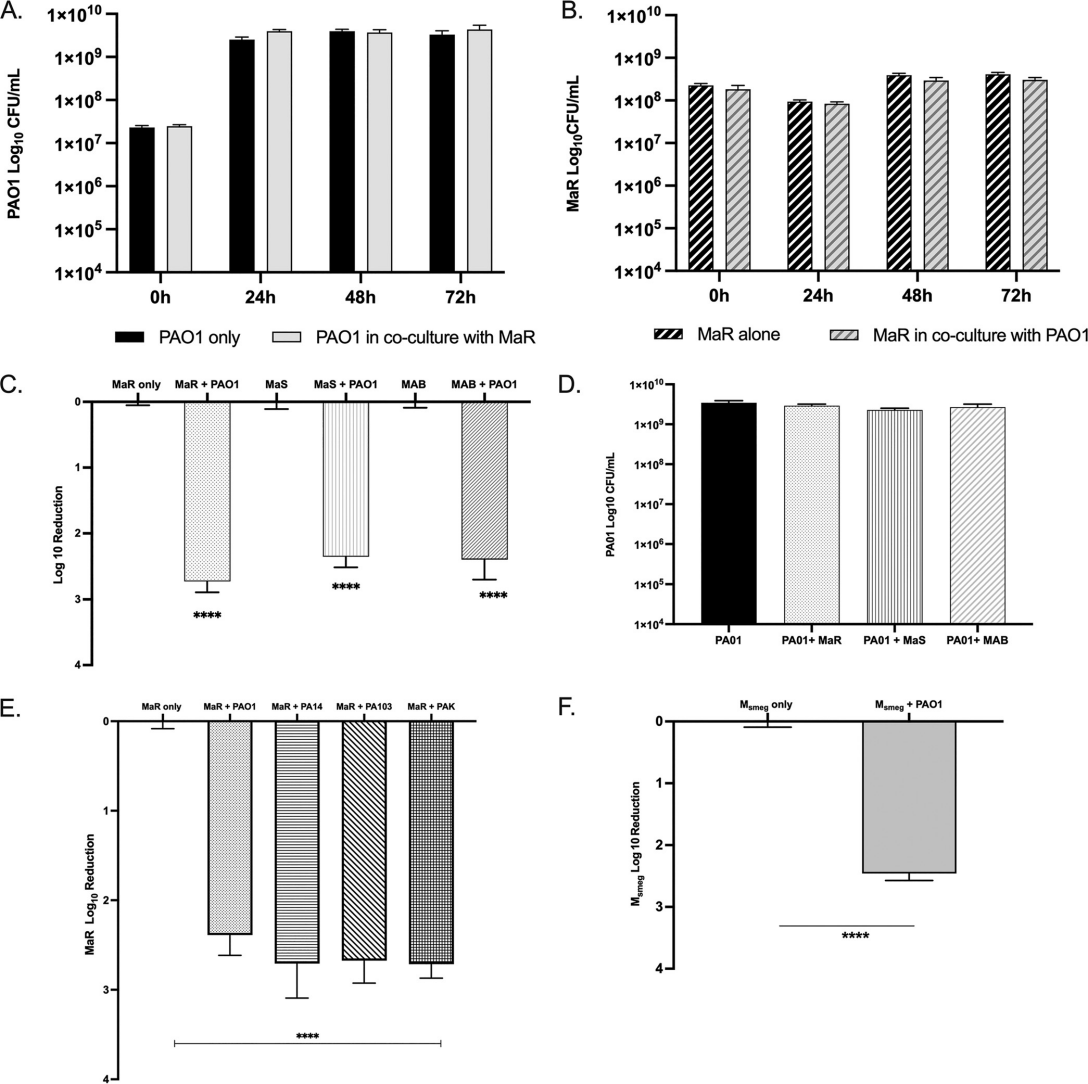
(A) Log_10_ CFU graph of PAO1 grown alone versus in planktonic coculture with MaR for 72 h. There was no statistical significance between the two conditions. (B) Log_10_ CFU/mL of MaR grown alone versus in planktonic coculture with PAO1 for 72 h (MaS data is found in Fig. S1A in the supplemental material). There was no statistical significance between the two conditions. (C) Log_10_ reduction CFU graph of MaR, MaS, and MAB (ATCC strain) grown in a single species biofilm compared to growth in a dual-species biofilm with PAO1. Unpaired *t* test; ****, *P* < 0.0001; *n* = 3. (D) Log_10_ CFU graph of PAO1 grown in a single-species biofilm compared to growth in dual-species biofilm with MaR, MaS, or MAB. (E) Log_10_ reduction graph of MaR grown alone compared to growth with PAO1, PA14, PA103, and PAK. Unpaired *t* test; ****, *P* < 0.0001; *n* = 3. (F) Log_10_ reduction graph of Mycobacterium smegmatis in a single-species biofilm compared to growth in dual-species biofilm with PAO1. Unpaired *t* test; ****, *P* < 0.0001; *n* = 3. Three biological replicates with two technical replicates were completed for each CFU study, except for that in panel F with two biological and technical replicates. All biofilm data represent day 4 CFU.

To initially examine the interaction between P. aeruginosa and M. abscessus, we utilized agar plates and liquid coculture. Across all time points analyzed for the liquid coculture, there was no difference in planktonic growth yields of either P. aeruginosa (Fig. 1A) or M. abscessus (Fig. 1B). This was consistent for both the rough and smooth M. abscessus variants (see Fig. S1A in the supplemental material). This indicated that when grown in liquid coculture there is no antagonism between the two organisms. We hypothesized that the antagonism between the two organisms might be density and/or lifestyle dependent and further investigated this using a colony biofilm model (8, 19).

To account for the aggregative nature of M. abscessus, liquid cocultures of MaR and PAO1 were grown in either 7H9 + Tween or 7H9 medium (Fig. S1B and C). No antagonism was observed in either medium. The change in the growth of MaR compared to that of PAO1 in liquid monocultures shows a visible optical density (OD) change over 72 h (see Fig. S4B in the supplemental material). We further considered the difference in growth rates between the organisms. Liquid cocultures with various initial suspension of P. aeruginosa PAO1 (optical density at 600 nm [OD_600_], 0.05, 0.150, and 0.5) were cocultured with M. abscessus MaR at an OD_600_ of 0.150 and showed that there is no significant difference in growth of MaR when in coculture with PAO1 at different starting ODs (Fig. S1D and E). Similarly, when bacteria were suspended in different volumes of media, no statistical difference was observed in M. abscessus growth (Fig. S1F and G).

To interrogate growth in biofilms, M. abscessus and P. aeruginosa dual-species biofilms were grown for 4 days on membrane filters placed on 7H10 medium at a multiplicity of infection (MOI) of 10:1 (19). Biofilm biomass and survival of each organism in dual-species biofilms were quantified by CFU and compared to growth in single-species colony biofilms. Both variants of M. abscessus were used, as they readily form biofilms (6). Interestingly, there was a significant decrease in M. abscessus abundance when grown in dual-species biofilms with P. aeruginosa compared to that of as M. abscessus single-species biofilm (Fig. 1C; note log scale). Antagonism was observed with each M. abscessus morphotype and the ATCC 19977 reference strain (MAB), which is a mixture of ~30% rough (MaR) and 70% smooth (MaS) morphotypes and the source of our isolated morphotypes (Fig. 1C). In contrast, there was no statistical significance in P. aeruginosa growth in dual-species biofilms compared to single-species biofilms (Fig. 1D), suggesting a unidirectional antagonism in biofilms.

P. aeruginosa must be viable for antagonism in dual-species biofilms since heat-killed P. aeruginosa and M. abscessus MaR do not show an antagonistic interaction (see Fig. S2A in the supplemental material). We also evaluated the role of the membrane filters in antagonism by spotting mono- and dual-species bacterial suspensions of M. abscessus and P. aeruginosa onto 7H10 agar, which also resulted in an antagonism of M. abscessus by P. aeruginosa (Fig. S2B). To further account for the difference in growth between the two bacteria, in addition to the MOI of 10:1 ratio of M. abscessus to PAO1, biofilms grown at MOIs of 1:1 and 100:1 resulted in the antagonism of M. abscessus (Fig. S1C and D). Remarkably, antagonism was also observed in experiments with three other P. aeruginosa strains (PA14, PA103, PAK) (Fig. 1E) and with another NTM bacterium, Mycobacterium smegmatis (Fig. 1F), showing that the antagonistic interaction is evident in multiple P. aeruginosa strains and against another NTM isolate.

### Temporal analysis of M. abscessus and P. aeruginosa interaction in biofilm.

**FIG 2:**
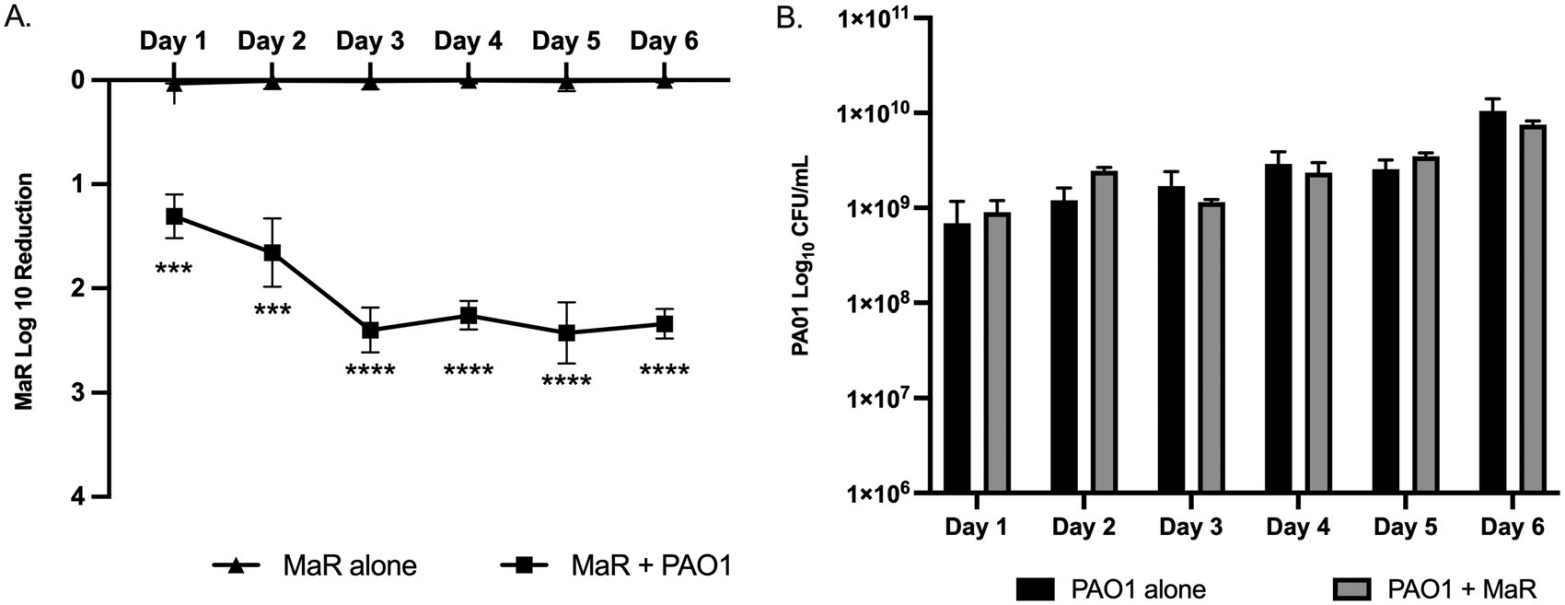
(A) Log_10_ reduction graph of kinetic biofilm over 6 days comparing MaR growth in single-species biofilm to dual-species biofilm with PAO1. Unpaired *t* test; ***, *P* < 0.001; ****, *P* < 0.0001; *n* = 3. Two filters were placed on each plate with three biological replicates. A similar log reduction was observed with the smooth morphotype (see Fig. S2E in the supplemental material). (B) Log reduction CFU graph of kinetic biofilm over 6 days comparing PAO1 growth in single-species biofilm to dual-species biofilm with MaR. Two filters were placed on each plate with three biological replicates.

To investigate the viability of M. abscessus in coculture with P. aeruginosa, dual-species biofilms were evaluated over 6 days. MaR CFU decreased significantly at days 1 and 2 in the dual-species biofilm, compared to a single-species biofilm and remained stable through days 3 to 6 (Fig. 2A), while P. aeruginosa, unlike MaR, showed unchanged growth over the 6 days (Fig. 2B). The same results were obtained with the smooth morphotype (MaS) (see Fig.S2E in supplemental material).

Kinetic biofilm growth studies suggested that M. abscessus might be killed or its growth inhibited by P. aeruginosa when in biofilm cocultures. We hypothesized that M. abscessus viability, measured by CFU, would decrease steadily over 6 days when in a dual-species biofilm with P. aeruginosa. However, M. abscessus viability remained constant from days 3 to 6 (Fig. 2A).

### Antagonism against M. abscessus is not maintained in the supernatant recovered from single P. aeruginosa only or from mixed biofilms.

Since P. aeruginosa can secrete various factors to antagonize the growth of other microorganisms, we evaluated if the antagonism toward M. abscessus resulted from P. aeruginosa-secreted factors. M. abscessus biofilms were grown using an inoculum of M. abscessus resuspended in supernatants harvested from P. aeruginosa single-species biofilm or dual-species P. aeruginosa and M. abscessus biofilms. There was a slight decrease in growth of MaR when resuspended in supernatant harvested from P. aeruginosa-only biofilm (Fig. S2F), but there was no significant difference in growth when resuspended in dual-species biofilm supernatant (Fig. S2G). In the case of MaS, there was no significant difference in growth when resuspended in MaS only and dual-species biofilm supernatant (Fig. S2H and I).

**FIG 3:**
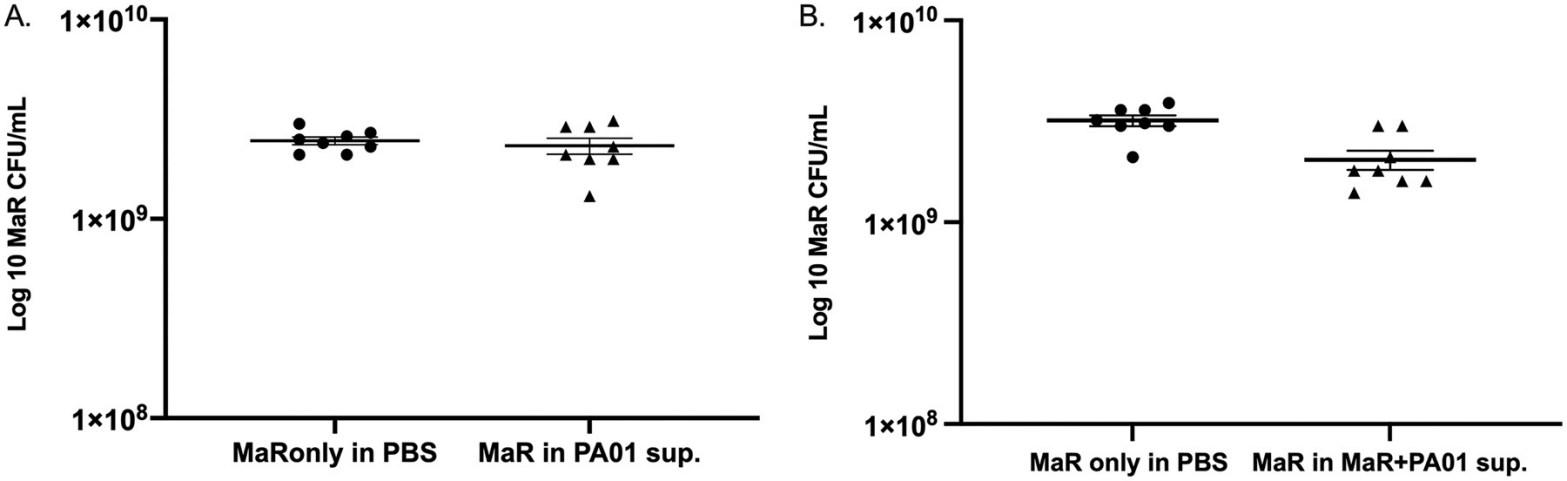
(A) Log_10_ CFU graph of MaR grown in 10-fold concentrated supernatant harvested from PAO1 biofilm. There is no statistical significance; mean +/− standard error of the mean (SEM) (*n* = 2); one technical replicate and two biological replicates. (B) Log_10_ CFU graph of MaR grown in concentrated supernatant harvested from PAO1 and MaR biofilm. There is no statistical significance; mean +/− SEM (*n* = 1); one technical replicate and two biological replicates.

We considered the possibility that P. aeruginosa-secreted factors might be diluted, as the biofilms were harvested into 5 mL of phosphate-buffered saline (PBS). Hence, we concentrated the supernatants 10-fold; however, this did not recapitulate the antagonism, as no significant difference in the survival of the M. abscessus rough morphotype resulted when grown in either P. aeruginosa-only supernatant (Fig. 3A) or supernatants harvested from P. aeruginosa and M. abscessus dual-species biofilms (Fig. 3B). These results suggested that the antagonism is surface dependent and requires cell-cell contact between P. aeruginosa and M. abscessus.

### Known P. aeruginosa contact-independent antagonism pathways are not responsible for M. abscessus antagonism.

**FIG 4:**
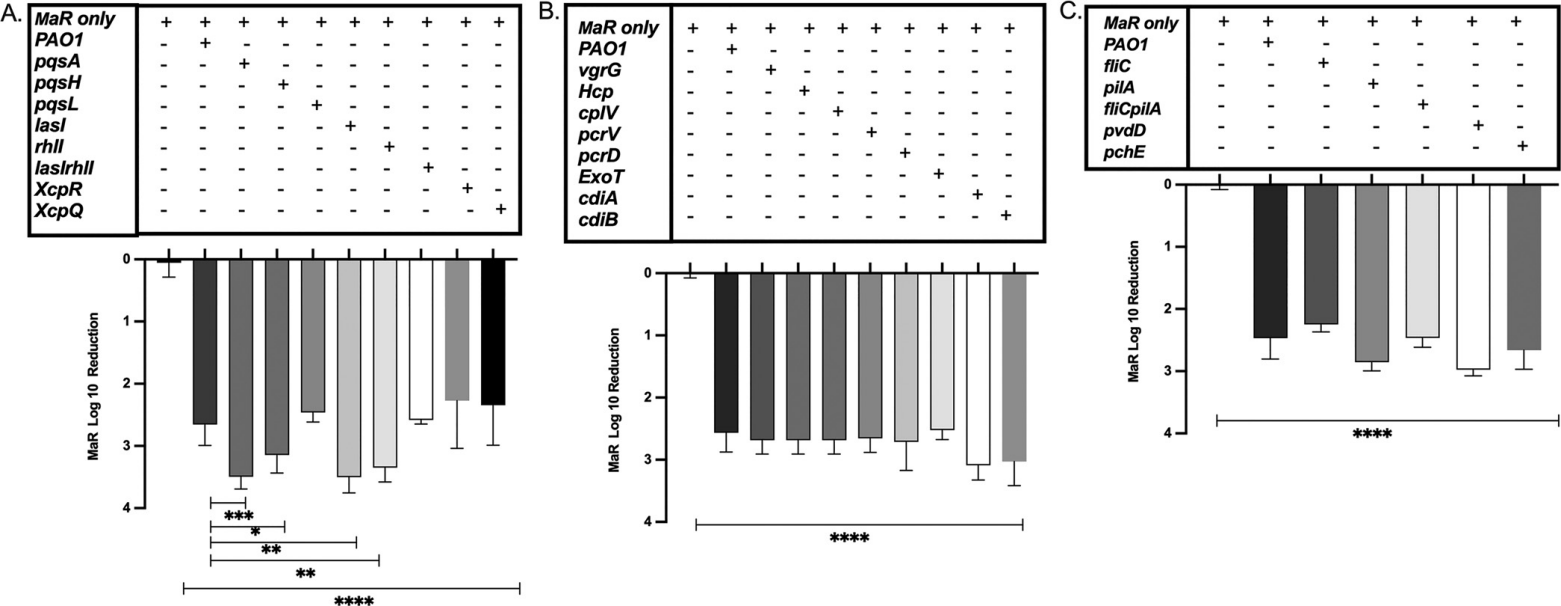
(A) Log_10_ reduction graph of MaR growth compared to that of P. aeruginosa QS (*lasI*, *rhlI*, and *lasI rhlI* mutants), PQS biosynthesis mutants (*pqsA*, *pqsL*, and *pqsH* mutants), and T2SS mutants (*xcpR* and *xcpQ* mutants) (*n* = 3). Unpaired *t* test; ****, *P* < 0.0001. Unpaired *t* test comparing PAO1 to *pqsA*, *pqsH*, *lasI*, and *rhlI* mutants; ***, *P* = 0.0001; *, *P* = 0.0121; **, *P* = 0.0028; and **, *P* = 0.0084, respectively. (B) Log_10_ reduction graph of MaR growth compared to that of P. aeruginosa T6SS (*vgrG*, *Hcp*, and *cplV* mutants), T3SS (*pcrV*, *pcrD*, and *exoT* mutants), and CDI mutants (Δ*cdiA_PA0041_* and Δ*cdiB_PA2463_* mutants) (*n* = 3). Unpaired *t* test; ****, *P* < 0.0001. (C) Log_10_ reduction of MaR growth compared to that of P. aeruginosa motility mutants (*fliC*, *pilA*, and *fliC pilA* mutants) and iron sequestering mutants (*pvdD* and *pchE* mutants) (*n* = 3). Unpaired *t* test; ****, *P* < 0.0001. All experiments evaluated two technical replicates and three biological replicates. See Table S1 in the supplemental material for a list of mutants used in this study. MaS results can be found in the supplemental material (see Fig. S3A to C).

We next investigated the mechanism responsible for the P. aeruginosa-mediated antagonism of M. abscessus by testing well-known P. aeruginosa contact-independent mechanisms. We hypothesized that PQS might play a role in the observed antagonism, as it has been shown in other systems (20). We evaluated dual-species biofilms of M. abscessus and P. aeruginosa *pqsA*, *pqsH*, and *pqsL* mutants. Antagonism of M. abscessus when grown with each of the PQS mutants was similar to that of wild-type PAO1 (Fig. 4A). Since PQS production is regulated by other P. aeruginosa QS systems, we also tested P. aeruginosa *lasI* and *rhlI* QS mutants. The antagonism was maintained in dual-species biofilms of M. abscessus and the P. aeruginosa QS mutants (*lasI*, *rhlI*, and *lasI rhlI* mutants) (Fig. 4A). These results were consistent across both morphotypes of M. abscessus (see Fig. S3A in the supplemental material). These data indicate that QS does not play a role in the observed antagonism against M. abscessus. We further examined biofilm cocultures with MaR and P. aeruginosa type II secretion system (T2SS) mutants, *xcpQ* and *xcpR* mutants, to test for other secreted molecules (Fig. 4A). Antagonism was also maintained in coculture with T2SS mutants.

### Well-studied contact-dependent interbacterial antagonistic mechanisms do not mediate the antagonism of P. aeruginosa on M. abscessus.

As known contact-independent mechanisms did not appear to play a role in the antagonistic interaction between the two bacterial species, we next investigated contact-dependent bacterial systems. We tested mutants to evaluate whether the P. aeruginosa type VI secretion system (T6SS) and type III secretion system (T3SS), as well as the contact-dependent inhibition (CDI) system played a role in the observed antagonism. All P. aeruginosa T3SS, T6SS, and CDI mutants tested in dual-species biofilm cocultures with M. abscessus failed to reverse the antagonism against M. abscessus (Fig. 4B). These results were consistent for both morphotypes of M. abscessus (Fig. S3B).

### Neither motility nor iron sequestration are responsible for the antagonism of M. abscessus.

Since known contact-independent and -dependent antagonistic mechanisms of interbacterial interaction were not responsible for the observed antagonism of M. abscessus, we next evaluated other key mechanisms of interbacterial antagonism that have been reported for P. aeruginosa, including motility and iron sequestration (18). We tested dual-species biofilms with M. abscessus and the P. aeruginosa *fliC* and *pilA* motility mutants, which are responsible for swarming and twitching motility, respectively, as well as the *fliC pilA* double mutant. Interestingly, antagonism was still observed in the presence of the motility mutant (Fig. 4C) and was the same for both variants of M. abscessus (Fig. S3C). This finding suggested that the motility of P. aeruginosa is not required for antagonism against M. abscessus.

Finally, we further tested the two main iron sequestering mutants of P. aeruginosa, including pyoverdine (*pvdD*) and pyochelin (*pchE*) to examine if P. aeruginosa iron sequestration leads to delayed growth of M. abscessus and aids in the growth of P. aeruginosa. Antagonism of M. abscessus was maintained, however, with both P. aeruginosa iron-sequestering mutants (Fig. 4C).

To determine the interbacterial mechanism responsible for the observed antagonism between M. abscessus and P. aeruginosa, we tested various contact-independent and contact-dependent mechanisms of P. aeruginosa. Our results indicated that all of these tested systems along with motility and iron sequestration mutants do not appear to mediate the observed antagonism, suggesting a novel mechanism of interbacterial antagonism.

## DISCUSSION

Despite the increased prevalence of M. abscessus in CF and in other pulmonary infections and its common isolation with P. aeruginosa, there is little understanding of the interaction between these two opportunistic pathogens. Here, we examined the interaction between these organisms in liquid planktonic cultures and biofilm cocultures. To determine the mechanisms responsible for the observed antagonistic interactions between M. abscessus and P. aeruginosa, mutants of known contact-dependent and -independent antagonistic mechanisms of P. aeruginosa were examined. However, all P. aeruginosa mutant strains that we examined were unable to abrogate the antagonism against M. abscessus, suggesting that a novel or a more complex mechanism may play a role in the observed antagonism. One of the few reports in the literature that examines the interaction between P. aeruginosa and M. abscessus showed that M. abscessus has enzymes that can degrade PQS produced by P. aeruginosa (16). This study mainly focused on purified and isolated M. abscessus enzymes, while we tested the direct interaction between these organisms. Although no antagonism between M. abscessus and P. aeruginosa was observed in liquid cocultures, including in those tested at different starting bacterial concentrations to account for growth difference between M. abscessus and P. aeruginosa, significant antagonism was observed in dual-species biofilm cocultures, suggesting that the pathway responsible for the observed antagonism was surface associated and concentration dependent. Interestingly P. aeruginosa antagonism was also observed with another rapidly growing NTM, Mycobacterium smegmatis. M. smegmatis is considered a model organism for molecular genetics and biochemical studies for several *Mycobacterium* species as many of the genes encoded by M. smegmatis are conserved in other mycobacterial species.

We therefore tested multiple different contact-independent mechanisms of P. aeruginosa antagonism, including PQS and QS mutants. PQS is a product of one of the four QS pathways in P. aeruginosa and plays a role in virulence factor regulation, iron sequestration, cytotoxicity, and biofilm formation (20, 22). PQS and other QS pathways lead to the production of different virulence factors that benefit P. aeruginosa when in association with other microbes, such as Staphylococcus aureus (21). Therefore, in addition to PQS, we also tested 2-heptylhydroxyquinoline-*N*-oxide (HQNO), a metabolite that acts in respiratory electron transfer (20, 22–24). PQS, HQNO (*pqsL*), and all other QS mutants tested, however, did not play a role in the P. aeruginosa interaction with M. abscessus (Fig. 4A). We also tested another contact-independent system of P. aeruginosa, T2SS, through which various toxins are released to the target. We evaluated T2SS-secreted factors using *xcpQ* and *xcpR* mutants of P. aeruginosa (25), but the antagonism was retained.

We, therefore, considered that it was possible that secretory molecules not regulated by the known QS systems of P. aeruginosa might be mediating the antagonism. This entailed a closer evaluation of contact-dependent mechanisms of interbacterial interaction. Specifically, we evaluated T6SS and T3SS mutants. In both systems, a translocation apparatus delivers toxins into target cells (26–30). For example, P. aeruginosa can kill heterologous bacterial species, including Vibrio cholerae and *Acinetobacter* via T6SS (31), while the T3SS is known to promote virulence against host cells (32). We further examined the role of contact-dependent growth inhibition, which is a two-partner secretion pathway through which Gram-negative bacteria can deliver a toxic C terminus domain of the CdiA protein into neighboring cells when in contact (33). This phenomenon, first described in Escherichia coli, can provide an advantage in competitive coculture conditions (34). However, none of the tested mutants reversed the observed antagonism (Fig. 4B). One potential explanation for this result is that the complex cell wall structure of M. abscessus, composed of multiple peptidoglycans and mycolic acids (35), may reduce the effectiveness of contact-dependent mechanisms preventing delivery of these toxins.

Both motility and the ability to take up nutrients are important for bacteria to survive and compete in multispecies environments (18). P. aeruginosa can compete against nonmotile Agrobacterium tumefaciens by blanketing the bacterium in dual-species biofilms. In this study, the formation of smaller microcolonies and reduced blanketing was observed with a P. aeruginosa *pilA* mutant (17). We, therefore, examined the role of P. aeruginosa motility, hypothesizing that the lack of motility by M. abscessus would provide an advantage for P. aeruginosa against M. abscessus. However, in this case, motility did not appear to play a role in the P. aeruginosa antagonism against M. abscessus.

Finally, iron sequestration can also be advantageous in interbacterial interactions, as studies have shown that P. aeruginosa can use lysed S. aureus as an iron source (18). This again was not the case in our study, as the two major iron sequestration mutants tested, pyoverdine (*pvdD* mutant) and pyochelin (*pchE* mutant), also failed to reverse the antagonism against M. abscessus (Fig. 4C). Mycobacteria also have molecules with a high affinity for iron (mycobactins and exochelins) (36), but since the antagonism is not reversed in the presence of P. aeruginosa iron sequestration mutants, the antagonistic mechanism(s) at hand may be overriding the contribution of iron sequestration.

In summary, we report a novel antagonistic interaction between P. aeruginosa and M. abscessus that is observed only in biofilms and not in planktonic coculture, suggesting that the biofilm lifestyle and likely direct contact between P. aeruginosa and M. abscessus contribute to antagonism. Surprisingly, the well-known P. aeruginosa contact-dependent and -independent interbacterial mechanisms, as well as motility and iron sequestration, do not appear to play a role in this antagonism, suggesting a novel interbacterial strategy.

As most of the study is focused on investigating what pathways might be causing the antagonism by P. aeruginosa, future studies using transcriptome sequencing (RNA-Seq) and sequencing approaches will investigate M. abscessus in the interaction. The novel antagonistic pathway responsible for the observed antagonism is important to study the interaction between these two pathogens since they can be coisolated from the same environmental reservoirs and from individuals with respiratory disease including CF. A better understanding of the interaction, as well as providing new information regarding bacterial defense mechanisms and antibacterial targets, will help to assess potential outcomes of this microbial interaction and the effects it can have on the host.

## MATERIALS AND METHODS

### Bacterial strains and growth conditions.

All bacterial strains and 21 transposon mutants of P. aeruginosa that were used are listed in Table S1 in the supplemental material. The P. aeruginosa PAO1 strain and M. abscessus ATCC 19977 strain were used as wild-type strains. M. abscessus was obtained from American Type Culture Collection (ATCC) and grown as directed. The ATCC strain is a combination of about 30% of the rough morphotype (MaR) and 70% of the smooth morphotype (MaS). To select for M. abscessus when in coculture with P. aeruginosa, we used mCherry M. abscessus. For mCherry transformation, each morphotype was grown, and an mCherry cassette with kanamycin resistance (provided by Sarah Fortune, Harvard University) was electroporated into the M. abscessus strain (6). We combined mCherry reporter morphotype strains in the same ratio as the M. abscessus ATCC 19977 type strain.

A 7H9 (Thermo Fisher) broth medium containing 10% oleic acid-albumin-dextrose-catalase (OADC) (Hardy Diagnostics) and glycerol as well as a 7H10 (Thermo Fisher) agar medium containing 10% OADC and glycerol were prepared as directed. The 7H10 agar and 7H9 and 7H9 + Tween liquid media were used for biofilm and liquid cocultures, respectively, as both M. abscessus and P. aeruginosa can grow in both forms of media. To isolate for P. aeruginosa, we used Pseudomonas isolation agar (PIA) plates (M. abscessus does not grow) and 7H10 + kanamycin plates to isolate for M. abscessus (PAO1 does not grow) (see Fig. S4A in the supplemental material).

OD_600_ values and their equivalent CFU values used in this study are as follows: for M. abscessus, an OD_600_ of 0.150 is equivalent to 2 × 10^8^. OD_600_ values for PAO1 at 0.05, 0.150, and 0.5 are equivalent to 2 × 10^7^, 5 × 10^7^, and 2 × 10^8^, respectively.

### Liquid coculture.

Overnight cultures of P. aeruginosa and M. abscessus were diluted to OD_600_ values of 0.05 and 0.150, respectively (16). M. abscessus colonies were scraped, suspended, and diluted in 7H9 from a 7H10 plate supplemented with kanamycin (100 μg/mL). As seen in Fig. S2J in the supplemental material, growing overnight cultures of M. abscessus in 7H9 + Tween versus scraping a loop full of an agar and diluting to the desired OD results in antagonism. M. abscessus and P. aeruginosa were pelleted and resuspended in 2 mL of 7H9 and incubated at 37°C and 200 rpm for 72 h. At every 24-h time point, aliquots were serially diluted and enumerated for CFU. Both 7H9 without Tween and 7H9 + Tween media were each tested, and no statistical difference was observed between the media (see Fig. S1B and C in the supplemental material). One milliliter of medium was used to replenish the cultures every 24 h. Replenishing the cultures with 2 mL of fresh medium versus 1 mL also resulted in antagonism (Fig. S1F and G).

### Colony biofilm.

Overnight cultures of P. aeruginosa were diluted to an OD_600_ of 0.05. M. abscessus colonies from 7H10 supplemented with kanamycin (100 μg/mL) were scraped, suspended, and diluted in 7H9 to an OD_600_ of 0.150 at an MOI of 1:10, respectively. Mixed cellulose esters membrane filters (25 mm, 0.45-μm pore size; Millipore) were UV sterilized on both sides for 5 min and placed on 7H10 solidified medium. One hundred microliters of bacteria cultures, either M. abscessus alone, P. aeruginosa alone, or mixed at a 1:1 ratio, was pipetted onto the filters to cover the entire membrane and incubated at 37°C with 5% CO_2_ for 4 days. Filters were transferred to new 7H10 plates at day 2 of incubation. At day 4, each filter was harvested by scraping biomass, with a cell scraper, into 5 mL of PBS. Harvested biomass was serially diluted and enumerated for CFU on 7H10 agar supplemented with kanamycin (100 μg/mL) or PIA to quantify M. abscessus and P. aeruginosa, respectively. To prevent clumping, which would affect CFU values, harvested biofilm bacteria were vortexed with glass beads and diluted in 7H9 + Tween (6). There were two filters on every plate with two technical replicates and three biological replicates performed for both mCherry morphotypes of M. abscessus.

For the biofilm kinetic analysis, colony biofilms of M. abscessus and P. aeruginosa, either alone or mixed, were set up as above. Biofilms were grown for 6 days, with the filters transferred to new 7H10 plate every 2 days. Biofilm biomass was harvested and quantified for CFU as above for each day.

Heat killed biofilms were tested by heat killing P. aeruginosa at 80°C for 30 min and inoculating a colony biofilm in coculture with M. abscessus.

Biofilms were also set up in six-well plates without membrane filters. Three milliliters of 7H10 agar was poured into six-well plates and left at room temperature overnight. The following day, 50 μL of M. abscessus and P. aeruginosa monoculture and dual cultures were prepared using an OD_600_ of 0.150 and 0.05, respectively, and were spotted onto the agar. At day 4, the biofilm was scraped with 3 mL of PBS and cell scraper and enumerated for CFU.

### Colony biofilm supernatant isolation.

Mixed M. abscessus and P. aeruginosa as well as single P. aeruginosa biofilms were grown for 4 days. For each replicate, six filters were harvested into 20 mL of PBS as mentioned above and centrifuged at 2,100 × *g* for 10 min to pellet cells and obtain supernatant. The supernatant was collected and filter sterilized twice using a 0.22-μm syringe filter. The sterile supernatant was collected into 50-mL conical tubes, frozen at −80°C overnight and lyophilized for 2 days. The resulting powder is resuspended in 2 mL of PBS for a 10-fold concentration.

### M. abscessus growth in biofilm supernatant.

M. abscessus (OD_600_ of 0.150) was pelleted by centrifugation at 2,100 × *g* for 10 min, washed in 1 mL PBS, and resuspended in 1 mL of the lyophilized supernatant. As a control, M. abscessus pellet was resuspended in 1 mL of PBS. The M. abscessus biofilms were grown for 4 days. Biofilm biomass was harvested, serially diluted, and enumerated for CFU. The M. abscessus biofilms were also set up with unconcentrated supernatant (supernatant obtained from the initial harvest in 5 mL PBS).

### Data availability.

All data generated or analyzed during this study are included in this published article (and its supplemental information files).
