# Supplementary material for: Surface Dependent Inhibition of Mycobacterium abscessus by Diverse Pseudomonas aeruginosa Strains
Source: Microbiol Spectr. 2022 Nov 17;10(6):e02471-22. doi: 10.1128/spectrum.02471-22 (PMC9769778; doi:10.1128/spectrum.02471-22)
Supplement: Supplemental file 1 — Supplemental material. Download spectrum.02471-22-s0001.pdf, PDF file, 0.7 MB [file spectrum.02471-22-s0001.pdf]

SUPPLEMENTAL DATA

**Bacterial Strains**	**Locus Tag**	**Description / genotype**
pqsA	PA0996	pqsA-H05::lacZbp02q1
pqsH	PA2587	pqsH-F01::lacZbp03q3
pqsL	PA4190	pqsL-H11::lacZbp02q4
lasI		Deletion mutant in PAO1 background
rhlI		Deletion mutant in PAO1 background
lasI rhlI		Deletion mutant in PAO1 background
vgrG	PA0091	vgrG1-D02::phoAwp01q2
Hcp	PA0263	HcpC-E04::phoAwp04q3
cplV	PA0090	cplV1-F02::phoAwp02q4
cdiA	PA2462	PA2462-G12::lacZwp02q4
cdiB	PA2463	PA2463-A05::phoAwp03q4
pcrV	PA1706	pcrV-F02::lacZbp02q3
pcrD	PA1703	pcrD-A09::phoAwp01q1
exoT	PA0044	exoT-C09::lacZwp03
fliC	PA1092	fliC-G10::phoAwp05q2
pilA	PA4525	pilA-E01::lacZwp08q4
fliC pilA		Double mutant in the MPAO1 background (Parseak Lab)
pchE	PA4226	pchE-E06:: phoAw07q4
pvdD	PA2399	pvdD-E07::lacZbp02q4
xcpR	PA3013	xcpR-D09:: phoAwp03q3
xcpQ	PA3105	xcpQ-EO3:: phoAbp02q4

Table S1. *P. aeruginosa* mutant strain

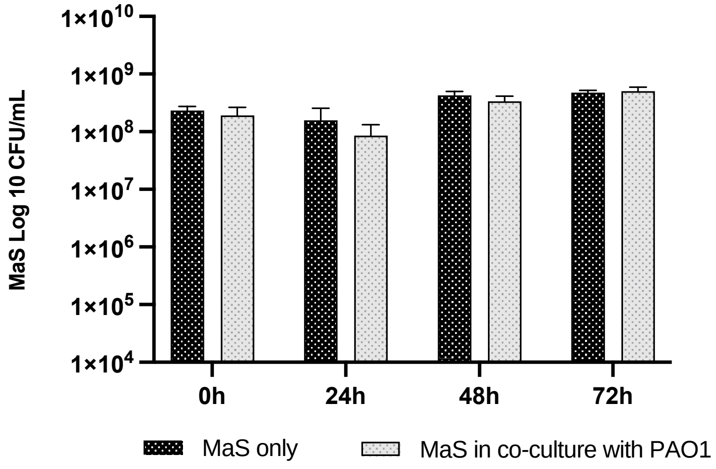
MaS Log 10 CFU/mL
1×10^{10}
1×10^{9}
1×10^{8}
1×10^{7}
1×10^{6}
1×10^{5}
1×10^{4}
0h
24h
48h
72h
MaS only
MaS in co-culture with PAO1

A.

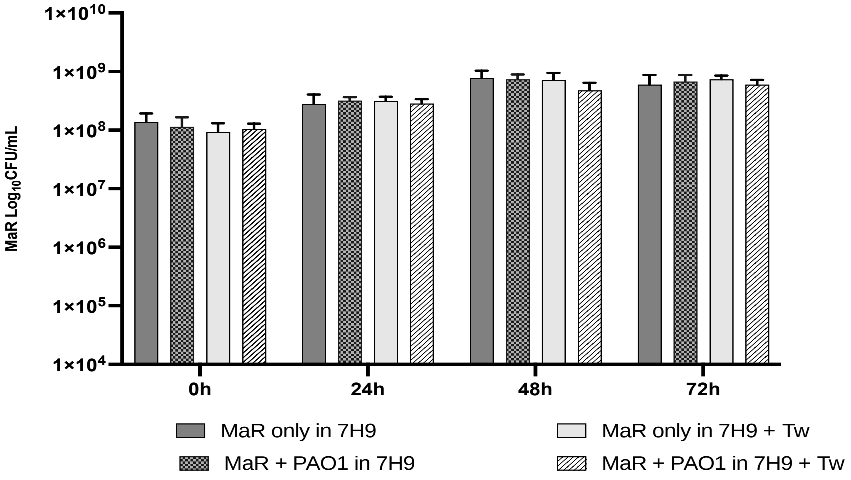
MaR Log_{10}CFU/mL
1×10^{10}
1×10^{9}
1×10^{8}
1×10^{7}
1×10^{6}
1×10^{5}
1×10^{4}
0h
24h
48h
72h
MaR only in 7H9
MaR only in 7H9 + Tw
MaR + PAO1 in 7H9
MaR + PAO1 in 7H9 + Tw

B.

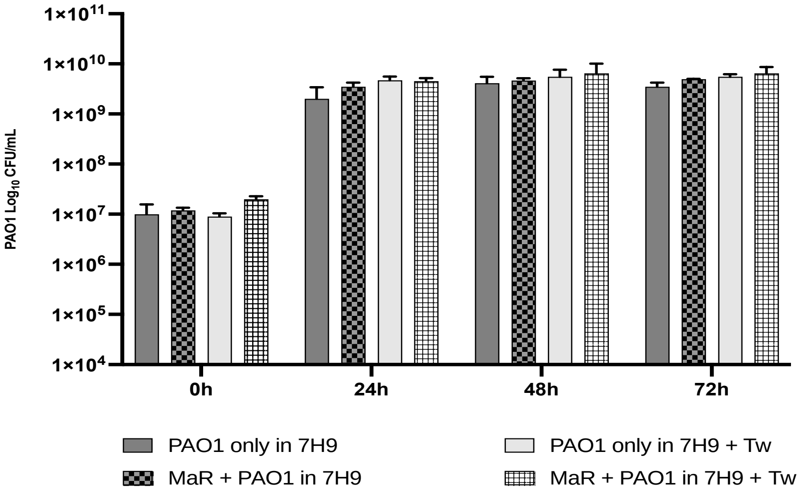
PAO1 Log_{10}CFU/mL
1×10^{11}
1×10^{10}
1×10^{9}
1×10^{8}
1×10^{7}
1×10^{6}
1×10^{5}
1×10^{4}
0h
24h
48h
72h
PAO1 only in 7H9
PAO1 only in 7H9 + Tw
MaR + PAO1 in 7H9
MaR + PAO1 in 7H9 + Tw

C.

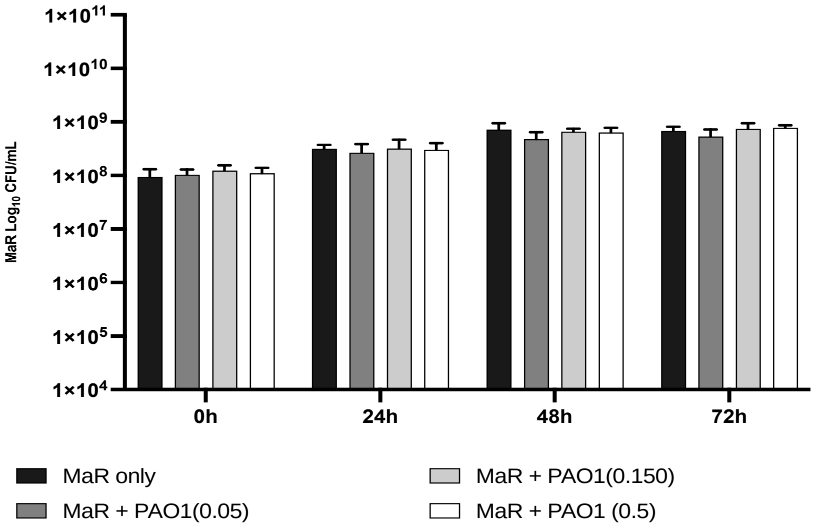
1×10^11
1×10^10
1×10^9
1×10^8
1×10^7
1×10^6
1×10^5
1×10^4
MaR Log10 CFU/mL
0h
24h
48h
72h
MaR only
MaR + PAO1(0.05)
MaR + PAO1(0.150)
MaR + PAO1 (0.5)

D.

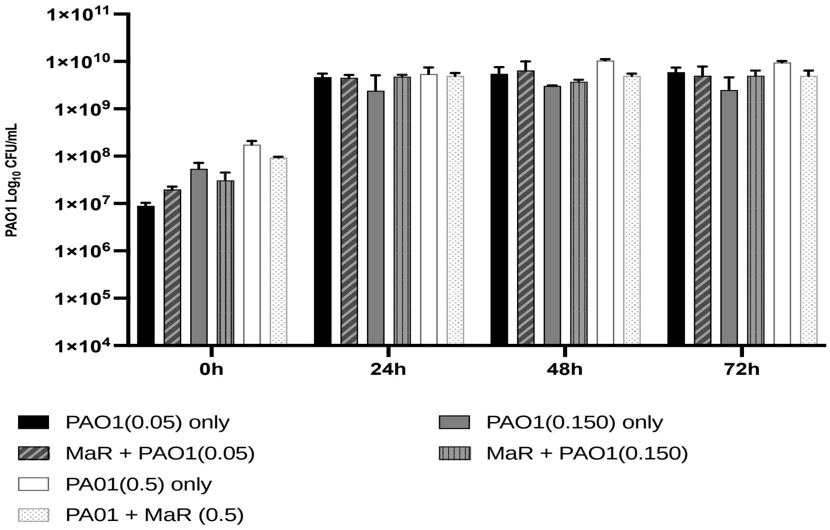
1×10^11
1×10^10
1×10^9
1×10^8
1×10^7
1×10^6
1×10^5
1×10^4
PAO1 Log10 CFU/mL
0h
24h
48h
72h
PAO1(0.05) only
MaR + PAO1(0.05)
PA01(0.5) only
PA01 + MaR (0.5)
PAO1(0.150) only
MaR + PAO1(0.150)

E.

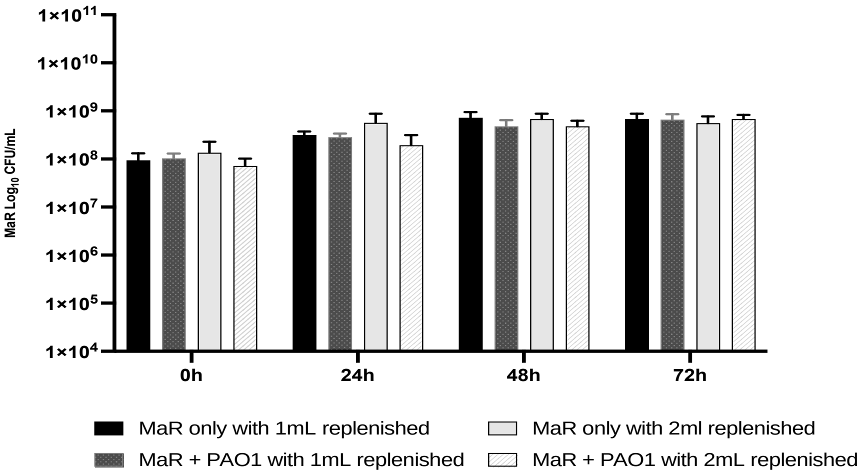
1×10^11
1×10^10
1×10^9
1×10^8
1×10^7
1×10^6
1×10^5
1×10^4
MaR Log10 CFU/mL
0h
24h
48h
72h
MaR only with 1mL replenished
MaR + PAO1 with 1mL replenished
MaR only with 2ml replenished
MaR + PAO1 with 2mL replenished

F.

G.

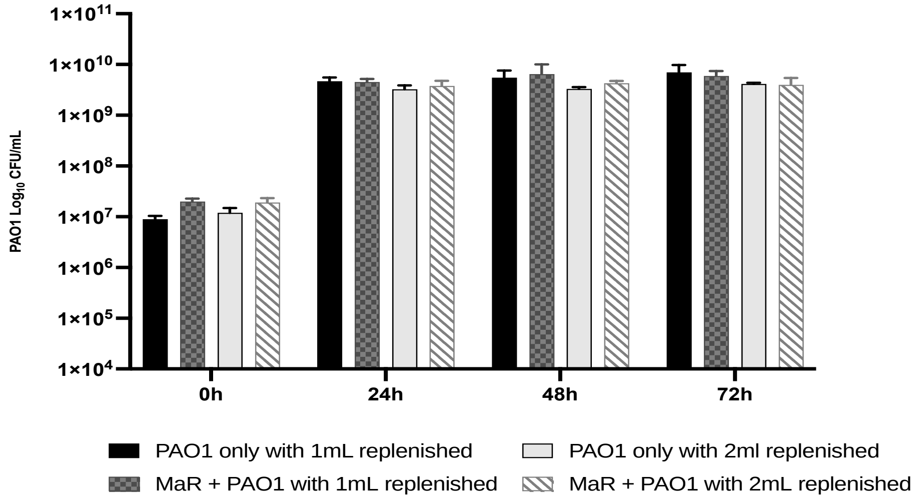

Figure S1. (A) Log_{10} CFU/mL of MaS grown alone versus in co-culture with PAO1 for 72 hrs. There was no statistical significance between the two conditions. (B) Log_{10} CFU/mL of MaR grown alone versus in co-culture with PAO1 for 72 hrs. in 7H9 vs 7H9 + Tween. There was no statistical significance between the two conditions. (C) Log_{10} CFU/mL of PAO1 grown alone versus in co-culture with MaR for 72 hrs. in 7H9 vs 7H9 + Tween. There was no statistical significance between the two conditions. (D) Log_{10} CFU/mL of MaR at OD_{600} of 0.150 grown alone versus in co-culture with PAO1 at different starting OD_{600} values of 0.05, 0.150 and 0.5 for 72 hrs. There was no statistical significance between the two conditions. (E) Log_{10} CFU/mL of PAO1 at different starting OD_{600} values of 0.05, 0.150 and 0.5 grown alone versus in co-culture with MaR at OD_{600} of 0.150 for 72 hrs. There was no statistical significance between the two conditions. (F) Log_{10} CFU/mL of MaR grown alone versus in co-culture with PAO1 for 72 hrs. with 1mL vs. 2mL media replenished every 24hrs. There was no statistical significance between the two conditions. (G) Log_{10} CFU/mL of PAO1 grown alone versus in co-culture with MaR for 72 hrs. with 1mL vs. 2mL media replenished every 24hrs. There was no statistical significance between the two conditions. N=2 for all data shown.

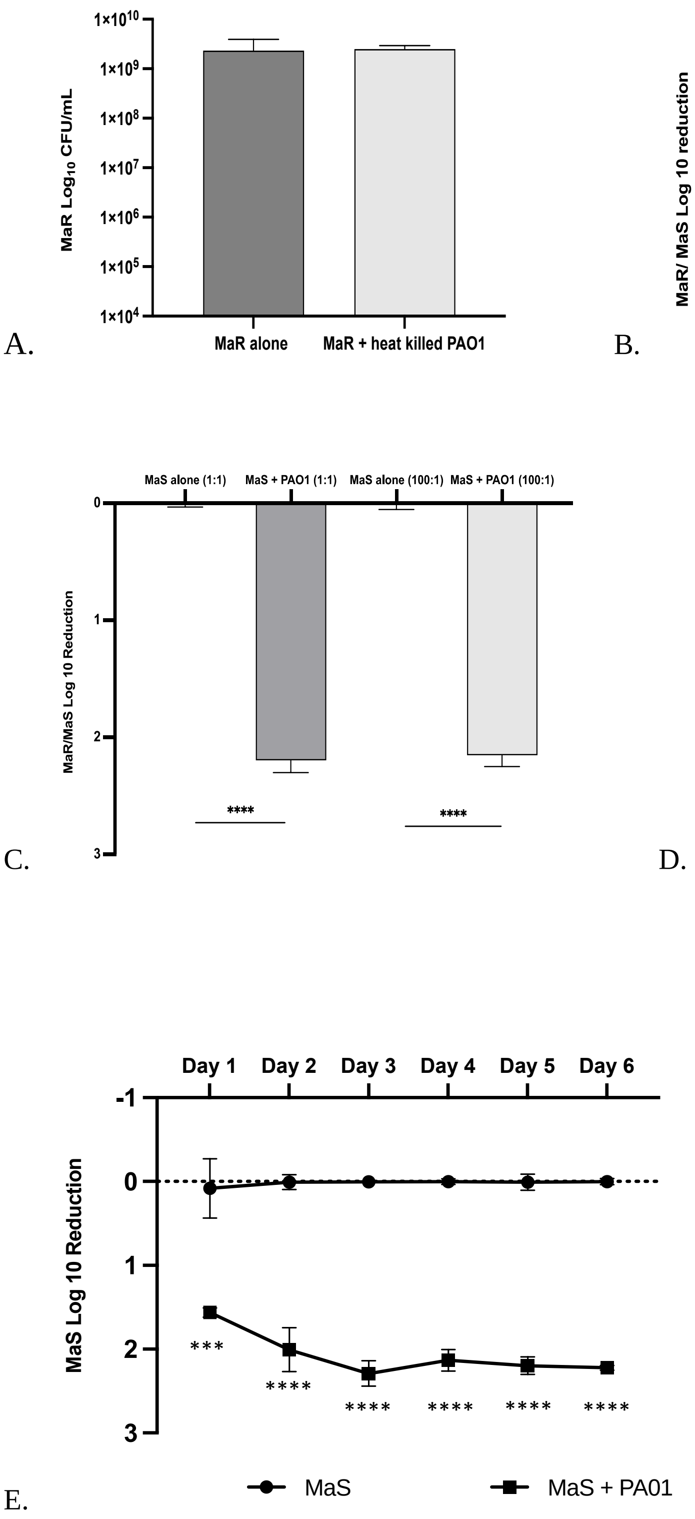
A.
MaR Log10 CFU/mL
1×10^10
1×10^9
1×10^8
1×10^7
1×10^6
1×10^5
1×10^4
MaR alone
MaR + heat killed PAO1
B.
MaS
MaS + PAO1
MaR
MaR + PAO1
MaR/ MaS Log 10 reduction
0
1
2
3
****
****
C.
MaS alone (1:1)
MaS + PAO1 (1:1)
MaS alone (100:1)
MaS + PAO1 (100:1)
MaR/MaS Log 10 Reduction
0
1
2
3
****
****
D.
MaR alone (1:1)
MaR + PAO1 (1:1)
MaR alone (100:1)
MaR + PAO1 (100:1)
MaR/MaS Log 10 Reduction
0
1
2
3
****
****
E.
Day 1
Day 2
Day 3
Day 4
Day 5
Day 6
MaS Log 10 Reduction
-1
0
1
2
3
***
****
****
****
****
****
MaS
MaS + PA01

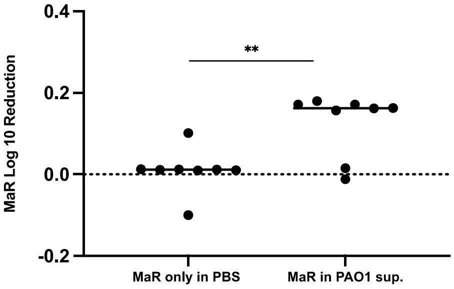
MaR Log 10 Reduction
0.4
0.2
0.0
-0.2
**
MaR only in PBS
MaR in PAO1 sup.

F.

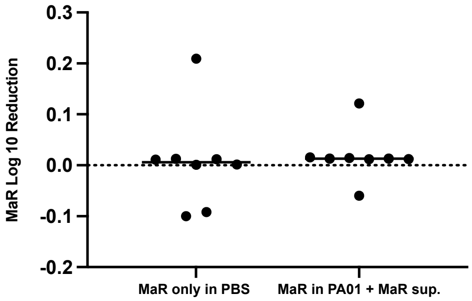
MaR Log 10 Reduction
0.3
0.2
0.1
0.0
-0.1
-0.2
MaR only in PBS
MaR in PA01 + MaR sup.

G.

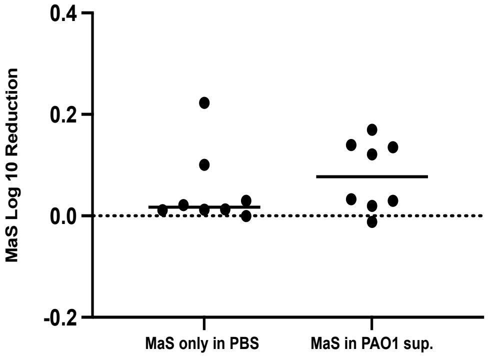
MaS Log 10 Reduction
0.4
0.2
0.0
-0.2
MaS only in PBS
MaS in PAO1 sup.

H.

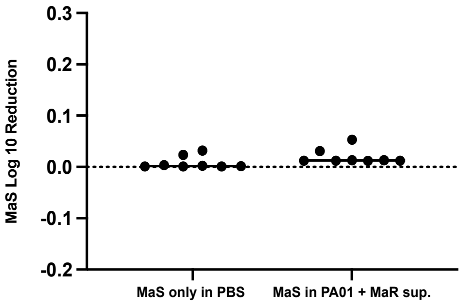
MaS Log 10 Reduction
0.3
0.2
0.1
0.0
-0.1
-0.2
MaS only in PBS
MaS in PA01 + MaR sup.

I.

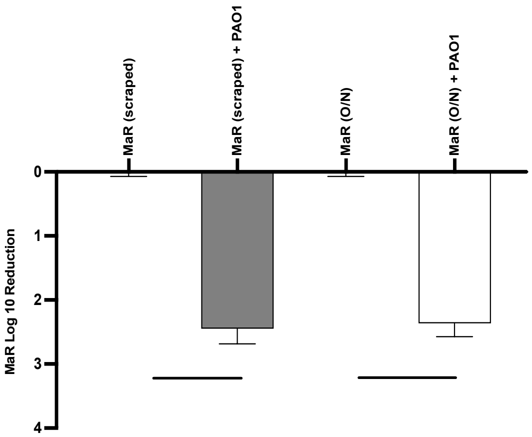
MaR (scraped)
MaR (scraped) + PAO1
MaR (O/N)
MaR (O/N) + PAO1
MaR Log 10 Reduction
0
1
2
3
4

J.

Figure S2. (A) Log_{10} CFU graph of heat-killed *P. aeruginosa* biofilm with MaR and MaS. Two filters were placed on each plate with two biological replicates. There was no statistical significance between the two conditions. (B). Log_{10} reduction graph of MaR, MaS with *P. aeruginosa* biofilm in six-well plate in the absence of a membrane filter. There were two technical and biological replicates for each condition (N=2). Unpaired t-test **** P<0.0001. (C) Log_{10} reduction graph of MaS with PAO1 biofilm at MOI of 1:1 and 100:1 respectively. Two filters were placed on each plate with two biological replicates (N=2). Unpaired t-test **** P<0.0001. (D) Log_{10} reduction graph of MaR with PAO1 biofilm at MOI of 1:1 and 100:1 respectively. Two filters were placed on each plate with two biological replicates (N=2). Unpaired t-test **** P<0.0001.(E) Log_{10} reduction CFU graph of kinetic biofilm over 6 days comparing *P. aeruginosa* growth in single-species biofilm to dual-species biofilm with MaS. Two filters were placed on each plate with three biological replicates. Unpaired T-test, *** P <0.001, **** P<0.0001 (N=2). (F) Log_{10} reduction graph of MaR grown in a single species biofilm compared to growth in the presence of diluted PAO1 supernatant (N=2). Unpaired t-test ** P=00034. (G) Log_{10} reduction graph MaR grown in a single species biofilm compared to growth in the presence of diluted PAO1 and MaR supernatant (N=2). There is no statistical significance. (H) Log_{10} reduction graph of MaS grown in a single species biofilm compared to growth in the presence of diluted PAO1 supernatant (N=2). There was no statistical significance. (I) Log_{10} reduction graph MaS grown in a single species biofilm compared to growth in the presence of diluted PAO1 and MaS supernatant (N=2). There was no statistical significance. (J) Antagonism of MaR was observed by either scraping an agar plate to dilute to the desired OD or preparing an overnight culture diluted to the desired OD. Two filters were placed on each plate with two biological replicates (N=2). Unpaired t-test **** P<0.0001.

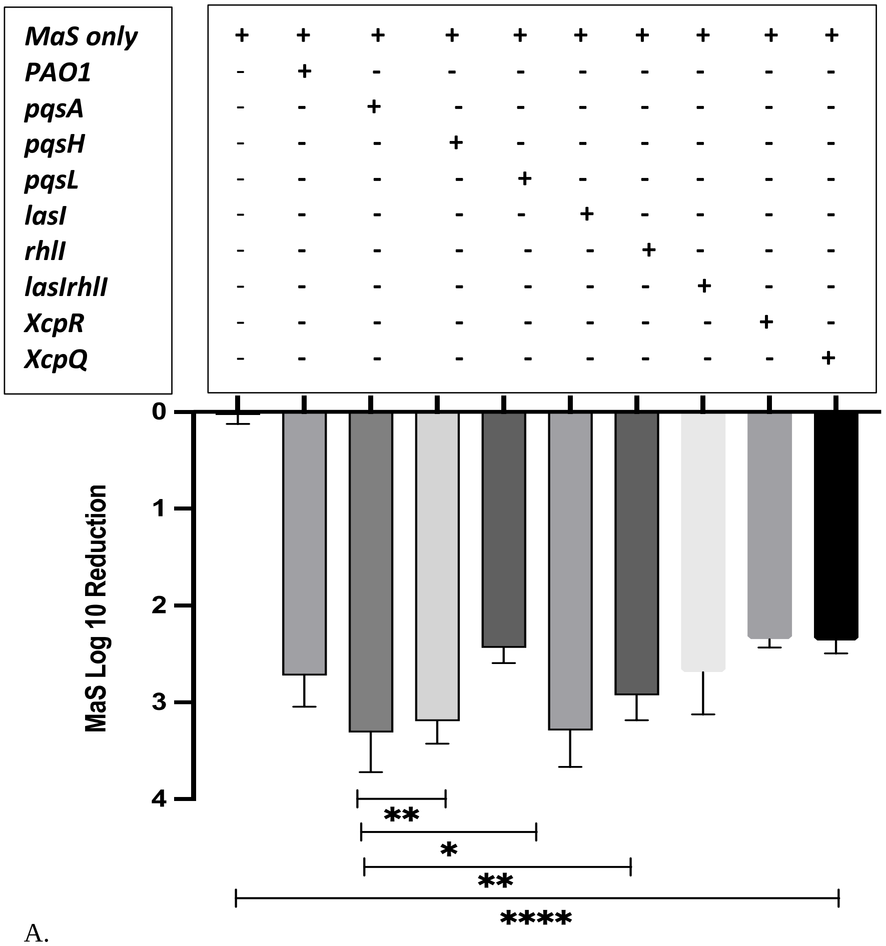
MaS only
PAO1
pqsA
pqsH
pqsL
lasI
rhlI
lasIrhlI
XcpR
XcpQ
MaS Log 10 Reduction
0
1
2
3
4
**
*
**
****

A.

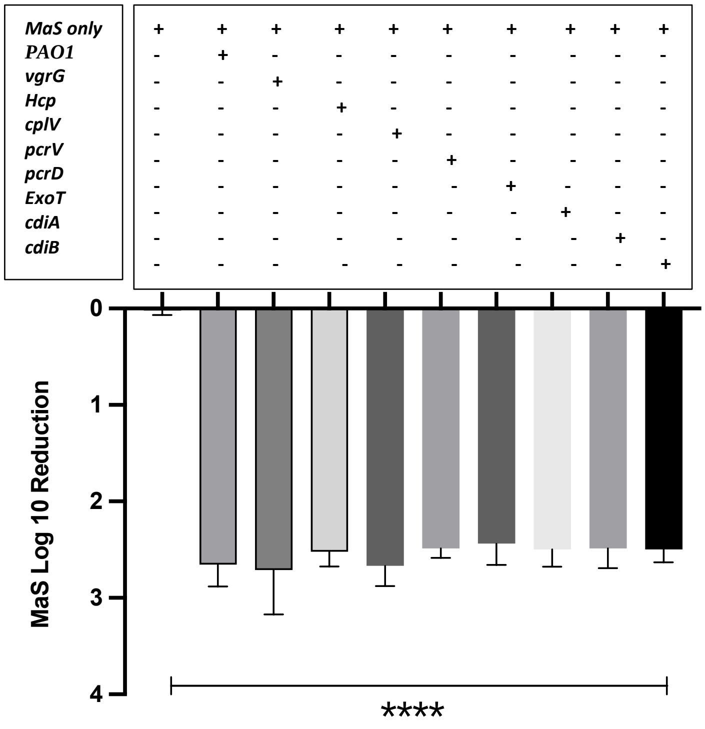
MaS only
PAO1
vgrG
Hcp
cplV
pcrV
pcrD
ExoT
cdiA
cdiB
MaS Log 10 Reduction
0
1
2
3
4
****

B.

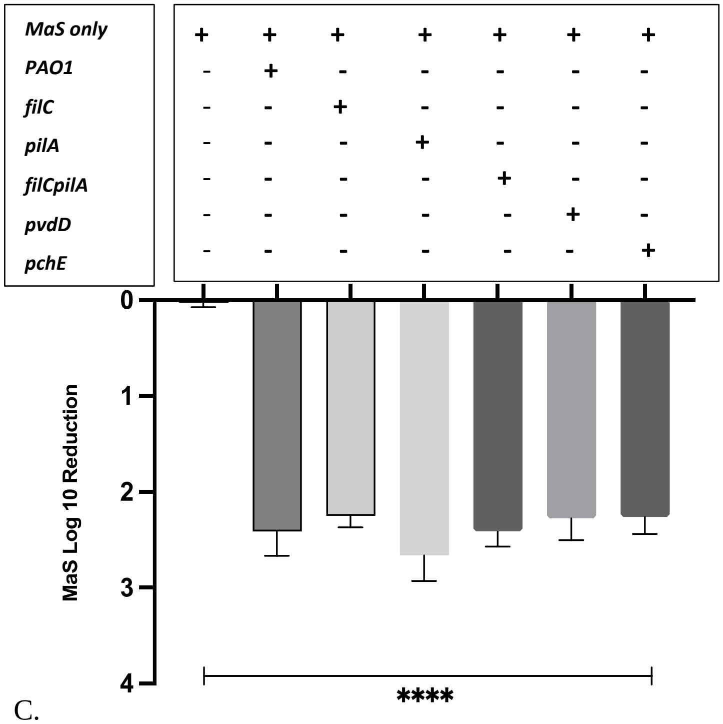
MaS only
PAO1
filC
pilA
filCpilA
pvdD
pchE
MaS Log 10 Reduction
0
1
2
3
4
****

C.

Figure S3. (A) Log_{10} reduction graph of MaS single-species biofilm growth compared to dual-species biofilm with *P. aeruginosa* QS (*lasI, rhlI, and lasIrhlI*), PQS biosynthesis mutants (*pqsA, pqsL, and pqsH*) and T2SS mutants (*xcpR* and *xcpQ*). (N=3). Unpaired t-test **** P<0.0001(Unpaired t-test comparing PAO1 to *pqsA, pqsH and, lasI* mutants* P=0.0112, ** P=0.0083, and *P=0.0105 respectively). (B) Log_{10} reduction graph of MaS single-species biofilm growth compared to dual-species biofilm with *P. aeruginosa* T6SS mutants (*vgrG, hcp, and cplV*), T3SS (*pcrV, pcrD, and exoT*) and CDI mutants ($\triangle cdiA_{PA0041}$ and $\triangle cdiB_{PA2463}$). (N=2). Unpaired t-test **** P<0.0001. (C) Log_{10} reduction of MaS single-species biofilm growth compared to dual-species biofilm with *P. aeruginosa* motility mutants (*fliC, pilA*, and *fliCpilA*) and iron sequestering mutants (*pvdD and pchE)* (N=2). Unpaired t-test **** P<0.0001. All experiments evaluated two technical replicates and two biological replicates except for with three biological replicates.

	**PAO1**	**MaR**	**MaS**
**PIA**	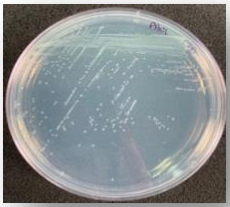	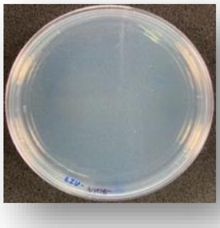	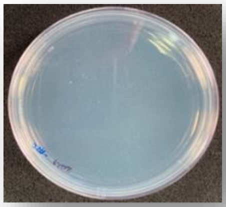
**7H10**	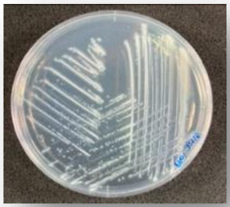	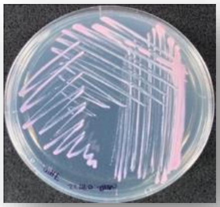	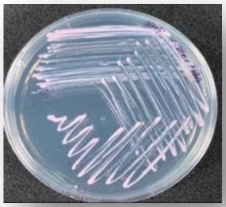
**7H10 + Kanamycin**	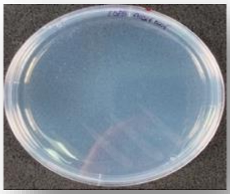	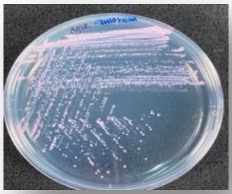	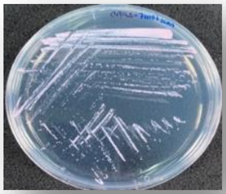

A.

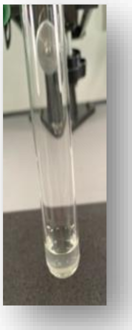 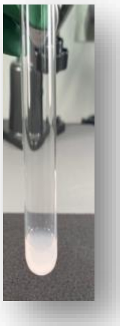 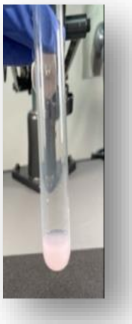 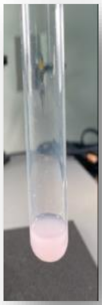

B

0h	24h	48h	72h

Figure S4. (A) MaS, MaR and PAO1 streaked on PIA, 7H10 plate and 7H10 + Kan plates. PIA plates incubated for 1 day since CFUs are counted on PIA plates are after 1 day of incubation and 7H10 and 7H10 + Kan plates incubated for 4 days. (D) Image showing the growth of MaR in liquid culture over 72hrs. Images were taken every 24hrs.
